# A Novel Organophosphorus Acid Anhydrolase from Deep Sea Sediment with High Degradation Efficiency for Organophosphorus Pesticides and Nerve Agent

**DOI:** 10.3390/microorganisms10061112

**Published:** 2022-05-27

**Authors:** Xiaofang Zheng, Li Wang, Lihong Qi, Zhiyang Dong

**Affiliations:** 1 State Key Laboratory of Microbial Resources, Institute of Microbiology, Chinese Academy of Sciences, Beijing 100101, China; 18745029415@163.com; 2 University of Chinese Academy of Sciences, Beijing 100049, China; 3 Department of Microbiology, College of Life Science, Hunan Normal University, Changsha 410081, China; 4 Institute of NBC Defense, PLA, Box1048, Beijing 102205, China; lihongqi8053@163.com

**Keywords:** organophosphorus compounds, organophosphorus acid anhydrolase, alkali-stable, salt-tolerant, biodegradation, bioremediation, deep-sea sediment

## Abstract

Organophosphorus compounds (OPCs), including highly toxic nerve agents and pesticides, have been used widely in agricultural and military applications. However, they have aroused widespread concern because they persistently pollute the environment and threaten human life. Organophosphorus acid anhydrolase (OPAA) is a promising enzyme that can detoxify OPCs. Here, a novel OPAA (OPAA114644) was isolated and characterized from deep-sea sediment (−3104 m). It exhibited excellent alkaline stability, and the loss of activity was less than 20% in the pH range 5.0–9.0, even after being incubated for 30 d at 4 °C. It also exhibited high salt tolerance, and its enzymatic activity increased by approximately fourfold in the presence of 20% NaCl (*w*/*v*). Additionally, OPAA114644 exhibited high degradation efficiency for soman, dichlorvos, paraoxon, coumaphos, and chlorpyrifos with a concentration of up to 250 mg/L, with the degradation rate being 100%, 100%, 100%, 80% and 51%, respectively, in 20 min under optimal conditions. Notably, OPAA114644 dissolved in different solutions, such as 20% NaCl, 1 mM SDS, 0.05% soap, 10% methanol, and tap water, could efficiently decontaminate the residual paraoxon on the surfaces of glasses, cotton tissues, and apples. These results indicate that OPAA114644 has excellent potential for the biodegradation and bioremediation of OPCs pollution and represents a real application of OPAA in the decontamination and detoxification of foods and clothes, and in the remediation of sites such as floors. Deep-sea sediment might also be an abundant resource for various functional microorganisms and enzymes.

## 1. Introduction

Organophosphorus compounds (OPCs) have been successively synthesized and applied by military forces as chemical warfare agents and in agriculture as pesticides since World War II [1]. Although the use of OP nerve agents as weapons in the military has been banned, terrorist attacks and assassination events caused by their misuse by terrorist organizations and extremists have occurred in recent years and caused serious harm [2]. The greatest concern regarding nerve agents is their high mortality, even at trace levels [3]. OP pesticides remain indispensable for maximizing crop yield in agriculture and account for over 38% of the total pesticides used worldwide [4]. Tens of thousands of tons of OP pesticides are used globally every year [5]; however, only 1% of the pesticides applied play a functional role, with the remainder entering water systems, leaking into the ground, and accumulating in the ecosystem because of their persistence, which results in environmental pollution that threatens human health [2,6]. According to the World Health Organization, three million OP pesticide poisonings occur every year, and 200,000 deaths are reported due to pesticide suicides and unintentional exposure [7].

Compared with traditional physical and chemical methods, enzymatic biodegradation is economical, efficient, and environmentally friendly [8], so this approach has attracted increasing attention. Several enzymes have been reported to degrade OPCs, such as the bacterial organophosphate hydrolase (OPH), methyl parathion hydrolase (MPH), organophosphate acid anhydrolase (OPAA), mammalian paraoxonase (PON), and squid diisopropylfluorophosphatase (DFPase) [8], among which the bacterial organophosphate hydrolase (OPH) and organophosphate acid anhydrolase (OPAA) are the most studied enzymes because of their high activity against OPCs. The characteristics of the organophosphorus-degrading enzymes mentioned above are listed in Table S1 [6].

OPH is also known as phosphotriesterase (PTE) and possesses a broad substrate spectrum compared to OPCs; however, OPHs exhibit a higher degradation efficiency for OP pesticides, particularly paraoxon and parathion, than for nerve agents [6]. By contrast, OPAAs exhibit a higher ability to degrade G-type nerve agents, such as sarin and soman, than OP pesticides. OPAAs from the *Alteromonas* species are one of the most studied enzymes and have been shown to be prolidases, which exhibit activity against both dipeptides with C-terminal proline (Xaa-Pro) and OPCs [9,10]. Prolidases are ubiquitous in nature and exist in eukarya, bacteria, and archaea. In eukarya, prolidases participate in protein degradation and collagen catabolism. The roles of prolidases in bacteria and archaea remain unclear, and their physiological function may involve protein metabolism rather than the detoxification of artificial OPCs [11]. The prominent feature of prolidases is that their protein structures consist of an N-domain and C-domain, and the C-domain exhibits a “pita bread” fold, which contains a dinuclear metal ion center coordinating two metal ions for activity [10,12]. Some other enzymes with the “pita bread” fold include aminopeptidase P, methionine aminopeptidase, dimethylsulfonioproprionate lyase, and creatinase, and they all have dinuclear metal ion centers, indicating that they may share a similar catalytic mechanism [13,14]. These enzymes, including prolidases, belong to the metallopeptidase family M24 [15].

The current research on organophosphate acid anhydrolase is mainly based on OPAAs from the *Alteromonas* species. Daczkowski et al. [16] used a structurally aided protein engineering approach to engineer the small pocket of OPAA from *Alteromonas* sp. JD6.5, which resulted in a 30-fold increase in catalytic efficiency toward racemic VR, with a strong stereospecificity toward the P(+) enantiomer-VR. Bae et al. [17] targeted the active sites of OPAA from *Alteromonas* sp. JD6.5 for site-directed mutagenesis and obtained the Tyr212Phe/Val342Leu (FL) mutant, which had a *k*_cat_/*K*_m_ value approximately six times higher than the wild-type (WT) enzyme. OPAA and the FL mutant were also used to develop biosensors for the detection of the OP pesticide paraoxon and G-type nerve agents [18,19]. Furthermore, a surface display of OPAA on the recombinant spore of *Bacillus subtilis* was constructed to significantly enhance resistance to various harsh conditions compared to free-form enzymes [20].

The Carlsberge Ridge in the Northwest Indian Ocean is one of the most complex spreading ocean ridges and has high-frequency hydrothermal vents. The deep-sea hydrothermal systems are highly rich in chemical substances such as Mn^2+^, Fe^2+^, H_2_, H_2_S, and methane [21,22]. Hot and chemically enriched fluid from the vent is mixed with surrounding cold seawater and forms a diversity of dynamic habitats with gradients of temperature and chemistry [21]. Therefore, distinct and unique microbial communities are formed, which contain abundant microbial and enzyme resources with unique characteristics. In addition, because of their remote and inaccessible location, it is challenging and not accessible to sample the deep-sea hydrothermal vents, so the sediment samples of deep-sea hydrothermal vents are invaluable, and it is significant and necessary to study the microbial and enzymatic diversity of these samples.

Considerable quantities of pesticides flow into rivers and finally converge in the ocean; therefore, the ocean may contain abundant resources of OP-pesticides-degrading microorganisms, and due to the special location of sediments in deep-sea hydrothermal vents below 3000 m, there may be potential enzyme resources with special properties. Because OPAA is considered a crucial component of enzyme-based OPCs decontamination cocktails, the Defense Threat Reduction Agency has called for the development of new enzymes that remain stable over broad ranges of temperature and pH, in the presence of salts and surfactants [23]. To develop new enzymes with efficient degradation potential for OPCs, a novel alkali-stable and salt-tolerant OPAA from deep-sea sediment was characterized and tested for its ability to degrade OPCs.

## 2. Materials and Methods

### 2.1. Gene and Chemicals

The deep-sea sediment, named Sample 13 (28I-(CR)-S021-TVG10), was collected from the hydrothermal vents of Carlsberge Ridge in the Northwest Indian Ocean (60°30′22.29″ E, 6°22′42.71″ N, −3104 m depth) with a television grab (TVG) device during the DY-125–28 cruise on 2 May 2013 by Lei Song and Jian Wang, Institute of Microbiology, Chinese Academy of Sciences. A fraction (10 g by wet weight) of the sediment sample was used for DNA extraction as described previously [22], and the metagenomic library was built and sequenced. The gene encoding OPAA114644 was obtained from the metagenomic library of the deep-sea sediment Sample 13 through a local Blastp search using the amino acid sequence of OPAA from *Alteromonas* sp. JD6.5 (AAB05590). Thereafter, the gene was codon-optimized for *E. coli* expression, synthesized, and cloned into the pET30a(+) plasmid by Shanghai Generay Biotech Co., Ltd. Subsequently, the recombinant plasmid was transformed into *E. coli* BL21 (DE3) strain (TransGen Biotech, Beijing, China) for protein expression. Demeton-S, dichlorvos, methyl-parathion, and malathion were purchased from ANPEL Laboratory Technologies Inc. (Shanghai, China). Coumaphos and paraoxon were purchased from Runzekang Biotechnology Co., Ltd. (Beijing, China). Diazinon was purchased from Separation Technology Co., Ltd. (Beijing, China). Chlorpyrifos was purchased from Sigma-Aldrich, Inc. (Darmstadt, Germany). Soman was obtained from Institute of NBC Defence, PLA. Gly-Pro, Leu-Pro, Met-Pro, Lys-Pro, Val-Pro, Phe-Pro, and Ala-Pro were purchased from Bankpeptide Biotechnology Co., Ltd. (Hefei, China). All other chemicals were of analytical reagent grade.

### 2.2. Protein Expression and Purification

For OPAA114644 expression, the BL21 (DE3) strain with recombinant plasmid was cultivated in 200 mL Luria-broth (LB) medium containing 50 µg/mL kanamycin and 0.2 mM MnCl_2_ at 37 °C. OPAA114644 expression was induced with 1 mM IPTG, and the protein was purified by nickel affinity chromatography with a 5 mL HisTrapTM HP column (GE Healthcare, Uppsala, Sweden as described previously [24]. The binding buffer was 50 mM Bis-Tris propane buffer, 0.5 M NaCl and 20 mM imidazole (pH 8.0), and the elution buffer was 50 mM Bis-Tris propane buffer, 0.5 M NaCl and 0.5 M imidazole (pH 8.0). A 5 mL HiTrapTM desalting column (GE Healthcare, USA) was used to remove imidazole with 50 mM Bis-Tris propane buffer (pH 8.0). The purified enzyme was verified by 12.5% SDS–PAGE, and the protein concentration was determined by Modified Bradford Protein Assay Kit (Sangon Biotech, Shanghai, China), with bovine serum albumin as the standard. To determine the oligomeric state of OPAA114644, its molecular weight in active state was measured by sedimentation velocity experiment using a ProteomeLab XL-I analytical ultracentrifuge (Beckman, Brea, CA, USA).

### 2.3. Degradation Efficiency of OPAA114644 for Different OP Pesticides and Nerve Agent

The degradation efficiency for different OP pesticides (paraoxon, parathion-methyl, diazinon, coumaphos, malathion, demeton-S, chlorpyrifos, and dichlorvos) and a nerve agent (soman) was tested. The reaction mixture contained 250 mg/L OP pesticides or nerve agent, 16.18 µM enzyme, and 0.2 mM MnCl_2_ in 50 mM Bis-Tris propane buffer (pH 8.0). The reaction mixture without enzyme was set as control. After the reaction mixture was incubated for 20 min at 50 °C, 1 mL mixture of 50% ethyl acetate and 50% n-hexane was added to extract the residual OPCs, which were applied in the GC-MS analysis using Q-TOF GC/MS (Agilent 7200, Agilent, Santa Clara, CA, USA). The carrier gas was highly pure helium at a rate of 3 mL/min. The split injector mode was used with a split ratio of 10:1, and the sample loaded was 10 µL at an inlet temperature of 290 °C. The initial temperature was 60 °C for 2 min, which was then increased to 290 °C at a rate of 15 °C/min and held for 10 min. The electron energy of electron ionization (EI) was 70 eV, and the source temperature was 230 °C. The residual OP pesticides and nerve agent were confirmed through the comparison of the hit ions of characteristic peaks in Agilent’s LC/MS database and spectral library.

### 2.4. Enzyme Assay

The organophosphorus acid anhydrolase activity against paraoxon was determined according to the described method [15]. The reaction mixture contained OPAA114644, 1 mM paraoxon, 0.2 mM MnCl_2_, and 50 mM Bis-Tris propane buffer (pH 8.0), with a total volume of 350 µL. The enzyme activity against paraoxon was determined on the basis of the production of p-nitrophenol at 405 nm by using a spectrophotometer. The data were logged every 30 s for 5 min at 50 °C. One unit of activity was defined as the amount of enzyme required to release 1 µmol p-nitrophenol per min at 50 °C. Paraoxon self-hydrolysis in the absence of enzyme was measured according to the aforementioned method.

The prolidase activity of OPAA114644 against several Xaa–Pro dipeptides (Val–Pro, Lys–Pro, Met–Pro, Phe–Pro, Leu–Pro, Ala–Pro, and Gly–Pro) was assayed through the measurement of proline released using the modified Cd-ninhydrin method [11]. The reaction mixture of 500 µL contained the enzyme, 4 mM Xaa-Pro dipeptide, 0.2 mM MnCl_2_, and 50 mM Bis-Tris propane buffer (pH 8.0) at 50 °C. The absorption value of the ninhydrin–proline complex was monitored at 515 nm. One unit of prolidase activity was defined as the amount of enzyme required to release 1 µmol proline per min at 50 °C.

Kinetic parameters of the enzyme against paraoxon and Met-Pro were determined by using several substrate concentrations (0.01–10 mM) according to the methods described above. Every experiment was performed in triplicate. GraphPad Prism 9.1.2 was used to calculate the kinetic constants according to the Michaelis–Menten equation.

### 2.5. Biochemical Characterization of OPAA114644

The optimal pH of OPAA114644 with paraoxon as substrate was measured in the following buffers at 50 °C: 50 mM acetic acid–sodium acetate (pH 5.0–6.2), 50 mM Bis-Tris HCl (pH 6.0–7.2), and 50 mM Bis-Tris propane (pH 7.0–9.2). To test pH stability, the enzyme was incubated in pH buffer ranges from 5.0 to 9.0 at 4 °C. Samples were taken at regular intervals, and the residual activity was measured under standard assay conditions, before which the pH was adjusted to 8.0. The optimal temperature was determined by testing the catalytic ability of the enzyme at 30–70 °C in 50 mM Bis-Tris propane buffer (pH 8.0). Thermostability experiments were performed through incubation of the enzyme at 30–60 °C (5 °C interval) for 15 min, 30 min, and 1 h, respectively, followed by rapid cooling in ice for 5 min, and the residual enzyme activities were calculated.

The effects of NaCl concentrations, organic solvents, and other chemical agents on OPAA114644 activity were measured by adding 10–30% (*w*/*v*) NaCl, 10% (*v*/*v*) organic solvents (methanol, ethanol, acetone, acetonitrile, and DMSO), and 1 mM or 0.1% (*v*/*v*) other agents (SDS, EDTA, DTT, Tween 20, Triton X-100, and β-mercaptoethanol) to the reaction mixture using 1 mM paraoxon as the substrate.

### 2.6. Metal Ions Analysis

Studies have indicated that the levels of PTEs’ activities were dependent on the presence of divalent cations in the growth medium [25,26]. Different divalent cations (0.2 mM MnCl_2_, CoCl_2_, ZnCl_2_, MgCl_2_, CuCl_2_, NiCl_2_, and CaCl_2_) were separately introduced into the growth medium to explore if the same phenomenon applies to OPAA114644. All the vessels used were washed with 30% nitric acid and then thoroughly rinsed with deionized water [26]. The medium and all the reagents were prepared with deionized water. After induction, cells with the same biomass were collected and broken. The activity of the supernatant against paraoxon after centrifugation was measured. Apoenzyme was prepared by incubating 1 mg/mL pure enzyme in 50 mM Bis-Tris propane buffer (pH 8.0) containing 50 mM EDTA and 1 mM β-mercaptoethanol for 48 h at 4 °C. Subsequently, the HiTrapTM desalting column was used to remove EDTA and β-mercaptoethanol with 50 mM Bis-Tris propane buffer (pH 8.0). Metal ions were reconstructed through the incubation of apoenzyme with 0.2 mM MnCl_2_, CoCl_2_, ZnCl_2_, MgCl_2_, CuCl_2_, NiCl_2_, and CaCl_2_, respectively, overnight at 4 °C. The enzyme activities against paraoxon were determined according to the aforementioned method.

To evaluate the effect of Mn^2+^ on the *T*_m_ of OPAA114644, differential scanning fluorimetry was performed to detect the denaturation temperature using NanoDSF (Prometheus NT.48, Nanotemper, Munich, Germany). The samples to be tested included the pure enzyme with or without 0.2 mM Mn^2+^ in the medium, the apoenzyme, and the apoenzyme reloaded with 0.2 mM Mn^2+^ with a final concentration of 1 mg/mL. The samples were loaded in 10 µL capillaries and scanned by increasing the temperature from 25 °C to 95 °C at a rate of 1 °C/min. The fluorescence values at 330/350 nm were logged, and the *T*_m_ values were detected.

### 2.7. Circular Dichroism Analysis

The circular dichroism of OPAA114644 was measured using a circular dichroism spectrometer (ChiraScan, Applied Photophysics, Leatherhead, Surrey, UK) at 20 °C. The protein concentration of the enzyme was 0.2 mg/mL, and the spectral signals were collected from 260 to 190 nm with a path length of 0.1 cm. The samples included the natural enzyme, the apoenzyme, and the apoenzyme reloaded with 0.2 mM Mn^2+^.

### 2.8. Decontamination of Paraoxon on Glasses, Cotton Tissues, and Apples by OPAA114644

#### 2.8.1. Glasses

To simulate the decontamination of pesticides on surfaces such as tables and floors, glasses (1 × 1 cm^2^) were contaminated with 10 nmol paraoxon in the fume hood, and left to stand for 10 min. Then, 2 mg of OPAA114644 was dissolved in 1 mL different solutions, including 50 mM Bis-Tris propane buffer (pH 8.0) with 20% NaCl, 1 mM SDS, 0.05% soap, 10% methanol or 10% ethanol, and tap water (pH = 7.1). Then, 10 µL of each enzyme solution was added to the glass surface for 10 min at room temperature. Residual paraoxon was extracted by 1 mL mixture of 50% ethyl acetate and 50% n-hexane, which was applied in the GC-MS analysis according to the description in Section 2.3.

#### 2.8.2. Cotton Tissues

To begin, 1 mg of OPAA114644 was dissolved in 10 mL of the different solutions described in Section 2.8.1. Cotton clothes were cut in squares (2 × 2 cm^2^) and contaminated with 0.5 µmol paraoxon in the fume hood. After 30 min, cotton tissues were incubated in each solution at room temperature. Aliquots of each solution were taken every 15 min to determine the production of p-nitrophenol at 405 nm [27]. Paraoxon self-hydrolysis in the absence of enzyme in different solutions was also measured. After washing, the cotton tissues were dried, and the residual paraoxon was extracted by a 2 mL mixture of 50% ethyl acetate and 50% n-hexane, which was applied in the GC-MS analysis. Different solutions (1 mL) were also taken out to extract the residual paraoxon by 1 mL mixture of 50% ethyl acetate and 50% n-hexane, which was applied in the GC-MS analysis.

#### 2.8.3. Apples

The decontamination of paraoxon on apples by OPAA114644 was performed according to the described method [27]. Apples (198 g) were contaminated with 1.5 µmol paraoxon in the fume hood. After 30 min, each apple was incubated in a solution of tap water containing 50 µg/mL OPAA114644. Aliquots of the washing solution were taken out at regular intervals to determine the production of p-nitrophenol at 405 nm. Two groups of controls were used. One group was a solution of tap water containing 1.5 µmol free paraoxon, which was set as positive control. The other group was a solution of tap water containing an apple contaminated with 1.5 µmol paraoxon in the absence of enzyme, which was set as control. After washing, the residual paraoxon was extracted from apples and washing solutions according to the description in Section 2.8.2 and applied in the GC-MS analysis.

## 3. Results and Discussion

### 3.1. Sequence Analysis

**Figure 1:**
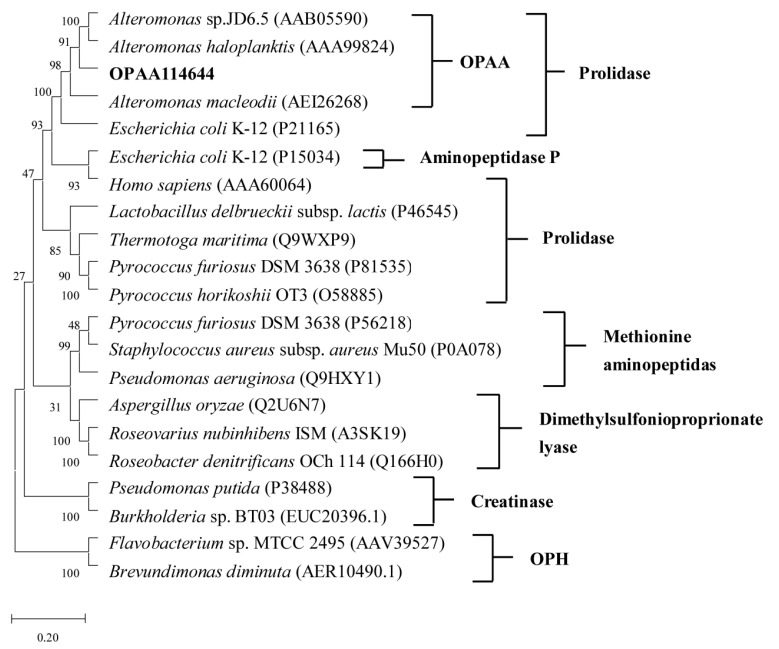
Neighbor-joining phylogenetic tree analysis of OPAA114644 based on the amino acid sequences. The tree was constructed using Mega5.0 software. The GenBank accession numbers of sequences are provided.

The OPAA114644 gene is composed of 1317 bp and encodes a protein of 438 amino acids. Blastp programs were used to search the non-redundant (nr) protein sequences database (https://blast.ncbi.nlm.nih.gov/Blast.cgi, accessed on 12 March 2022) using the OPAA114644 amino acid sequence, and the results revealed that the enzyme had the highest similarity (79.63%) with Xaa-Pro dipeptidase from *Gammaproteobacteria bacterium*, which has not been studied. To evaluate the evolutionary relationship of OPAA114644, a phylogenetic tree based on amino acid sequences was constructed (Figure 1). Bacterial PTEs such as the enzyme from *Brevundimonas diminuta* and OPAAs from *Altermonas* species have been well studied, and both can efficiently degrade OPCs. However, they are not homologous, suggesting that their function of OP degradation might have evolved from different ancestors. OPAAs/prolidases, creatinase, aminopeptidase P, methionine aminopeptidase, and dimethylsulfoniopropionate lyase perform different functions despite all belonging to metallopeptidase family M24 and sharing the same “pita bread” fold. According to the phylogenetic tree, OPAA114644 had a closer evolutionary link with OPAAs from *Altermonas* species and exhibited 61% similarity with OPAA from *Altermonas* sp. JD6.5.

**Figure 2:**
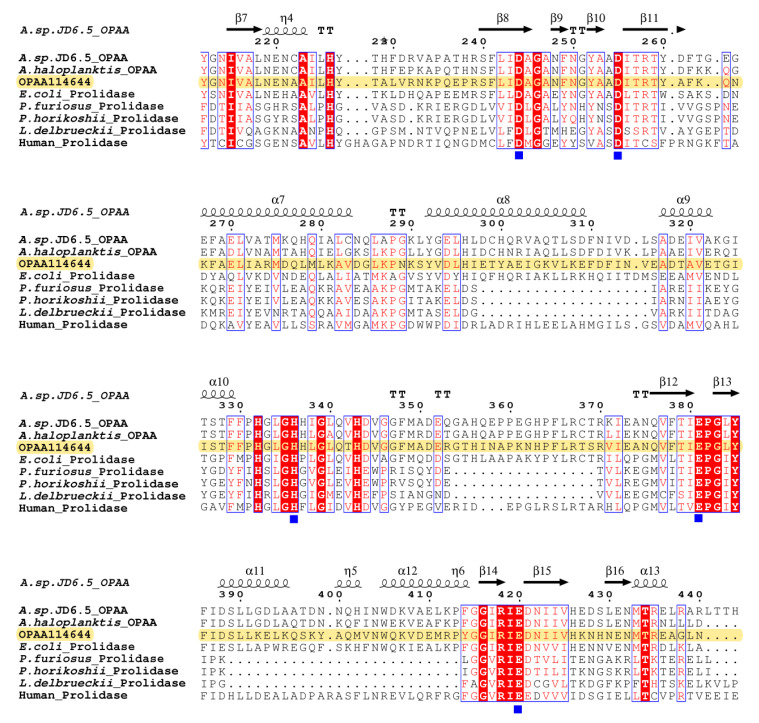
Multiple sequence alignment of OPAA114644 with other characterized prolidases. These enzymes were OPAAs from *Alteromonas* sp. JD6.5 (AAB05590) and *Alteromonas haloplanktis* (AAA99824) and prolidases from *E. coli* (P21165), human (AAA60064), *Lactobacillus delbrueckii* (P46545), *Pyrococcus furiosus* (P81535), and *Pyrococcus horikoshii* (O58885). The sequence of OPAA114644 was highlighted. The relatively conserved residues are indicated in red and boxed in blue, and the identical residues are marked with a red background. The five highly conserved amino acids coordinating with metal ions sites are marked with blue squares. The secondary structure of OPAA114644 was characterized based on the crystal structure of OPAA from *Alteromonas* sp. JD6.5 (PDB: 4zwo).

Multiple sequence alignment was performed using ClustalX and rendered using ESPript [28]. The predicted secondary structure of OPAA114644 was compared with those of other prolidases from different sources, and the results are presented in Figure 2. OPAA114644 has five highly conserved amino acids (Asp243, Asp254, His334, Glu379, and Glu418) in the C-terminal region, which were reported to bind metal ions for catalytic requirements in other prolidases. Despite differences in sources, metal ions, and oligomeric states, these prolidases possess the same five conserved residues [10,29,30]. For OPAA from *Alteromonas* sp. JD6.5, the five conserved amino acids are Asp244, Asp255, His336, Glu381, and Glu420. His336 and Glu381 participate in the ligands of one Mn^2+^ ion, while Asp244 solely coordinates with the other Mn^2+^ ion. Asp255 and Glu420 act as bidentate bridges between the two Mn^2+^ ions [10]. Similar patterns were observed in prolidase from *Pyrococcus furiosus* and methionine aminopeptidase from *E. coli* where the metal ions were Co^2+^ [31,32]. Therefore, it is predicted that the five highly conserved amino acids in OPAA114644 metal ions ligands are arranged in the aforementioned manner. Notably, several amino acids adjacent to the five metal ligand residues, such as Pro380, Gly381, Arg416, and Ile417, are also highly conserved in the C-terminal region (Figure 2), suggesting that they may play key roles in the catalytic reaction. Thus, the sequence analysis revealed that OPAA114644 has the potential to degrade OPCs and Xaa-Pro dipeptides.

### 3.2. Protein Expression and Purification

**Figure 3:**
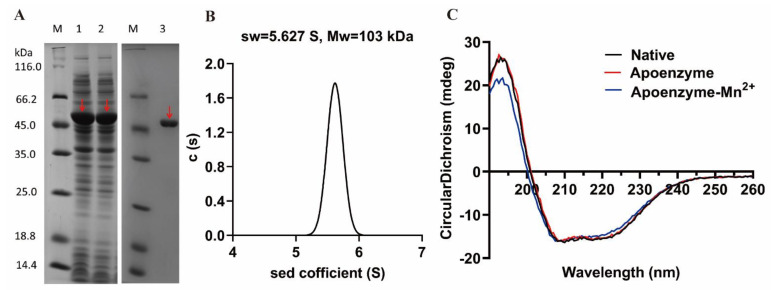
Protein expression and purification. (**A**) SDS-PAGE analysis of OPAA114644. Lanes: M, molecular weight marker; 1, whole-cell extract; 2, supernatant; 3, purified OPAA114644. OPAA114644 is indicated with red arrows. (**B**) Sedimentation velocity experiment. (**C**) Circular dichroism spectrum analysis. The black curve is native OPAA114644; red curve is apoenzyme; and blue curve is apoenzyme reconstructed with 0.2 mM Mn^2+^.

The recombinant OPAA114644 was successfully expressed by *E. coli* with a dominant band at approximately 51 kDa on the SDS-PAGE gel (Figure 3A). After purification and desalination, the yield of pure enzyme was 226 mg/L of culture, which was higher than that of the recombinant OPAA from *Altermonas* sp. JD6.5 (150–200 mg/L of culture) [33]. The high yield of this enzyme makes it favorable for large-scale production in practical application.

A sedimentation velocity experiment was performed to determine the oligomeric state of OPAA114644 in solution. The sedimentation coefficient was 5.627 S, and the molecular weight of purified recombinant OPAA114644 in solution was 103 kDa (Figure 3B), which was in accordance with the value for a dimer (2 × 51 = 102 kDa).

Circular dichroism spectrum analysis was performed to measure the secondary structure of OPAA114644. The circular dichroism spectrum of native OPAA114644 was typical α-helical with two peaks at 208 and 220 nm (Figure 3C).

### 3.3. Degradation Efficiency of OPAA114644 for Different OP Pesticides and Nerve Agent

**Figure 4:**
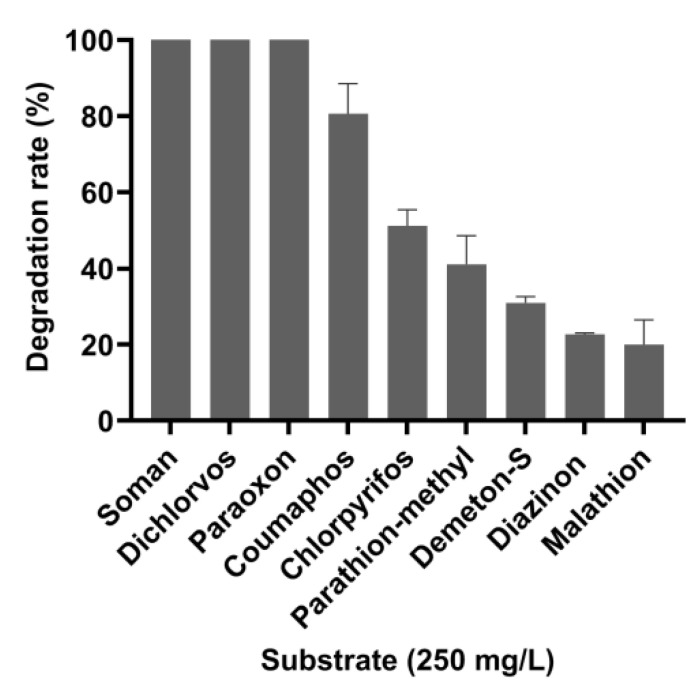
Degradation of OP pesticides and nerve agent by OPAA114644. The values are an average of three independent experiments, and error bars represent standard deviation.

OPAAs can degrade nerve agents and pesticides with P–F, P–CN, P–S, and P–O bonds. However, there are few reports available on the degradation of other pesticides by OPAAs, except for paraoxon. The degradation efficiency of OPAA114644 for eight OP pesticides, namely paraoxon, parathion-methyl, diazinon, coumaphos, malathion, demeton-S, chlorpyrifos, dichlorvos, and the nerve agent GD, were measured (Figure 4, Figure S1). OPAA114644 could completely degrade 250 mg/L soman, dichlorvos, and paraoxon in 20 min. Moreover, it could degrade 80% of coumaphos and 51% of chlorpyrifos. However, the degradation efficiency of OPAA114644 for parathion-methyl, demeton-S, diazinon, and malathion was less than 50%. According to the result, OPAA114644 had a greater affinity for degrading P–F and P–O bonds than P–S bond. A prolidase from *Pseudoalteromonas* sp. SCSIO 04,301 presented a similar substrate preference [15]. In addition to P–F, P–CN, P–S, and P–O bonds, OP compounds also contain a P=O or P=S bond (Figure S1). OPAA114644 exhibited a higher activity for pesticides with a P=O bond, such as paraoxon and dichlorvos, than for those with a P=S bond, such as coumaphos and chlorpyrifos. Although soman has been banned because of its high fatality rate, it is still being misused by terrorist organizations and extremists, representing a threat to public safety. Dichlorvos and chlorpyrifos are moderately toxic pesticides, and they are widely used to control various types of crop pests [3]. Coumaphos is a broad-spectrum insecticide and affects various ectoparasites of livestock; thus, it is extensively applied to eradicate ticks on farms. Paraoxon and parathion-methyl were formerly used to control various agricultural pests but are now banned because of their high toxicity; however, they still exist in the ecosystem. The high degradation efficiency of OPAA114644 for OP pesticides and nerve agents indicates its potential use for the detoxification and bioremediation of OPCs. The results also illustrate that deep-sea sediment might be an abundant resource for various functional enzymes.

### 3.4. Enzyme Assay

As prolidases, OPAAs exhibit activity against dipeptides with proline at the C-terminus. Experiments were performed to measure the enzyme activity of OPAA114644 on several Xaa-Pro dipeptides (Figure S2). OPAA114644 had different levels of activity for Xaa-Pro dipeptides (Met-Pro > Ala-Pro > Leu-Pro > Lys-Pro > Phe-Pro > Gly-Pro > Val-Pro), and the preferred Xaa-Pro dipeptide substrate was Met-Pro, with the specific activity being 3095 U/mg.

The kinetic parameters of OPAA114644 for Met-Pro and paraoxon were measured (Table 1, Figure S3). Although the *K*_m_ values of OPAA114644 for Met-Pro and paraoxon were not significantly different, other kinetic parameters for Met-Pro were three orders of magnitude higher than those for paraoxon, suggesting that Xaa-Pro dipeptides were the optimal substrate of this enzyme, other than OPCs.

### 3.5. Biochemical Characterization of OPAA114644

The pH profile of OPAA114644 was measured in the pH range of 5.0–9.2. OPAA114644 exhibited an alkali-active characteristic, with the optimal pH being 8.0 in 50 mM Bis-Tris propane buffer (Figure S4A). The enzyme activity increased sharply in the pH range 6.8–8.0 and maintained approximately 88% of the high activity at pH 9.2. An examination of enzymatic stability in different pH solutions confirmed the high alkali stability over a wide pH range (Figure S4B). The enzyme was stable, and the loss of activity was less than 10% and 20% compared with the respective initial activity in the pH range 5.0–9.0, even after being incubated for 16 and 30 days, respectively. The ability of OPAA114644 to maintain stability and activity over a wide pH range over an extended period makes it an excellent candidate for further application.

The OPAA114644 enzyme was mesophilic and exhibited an optimal temperature of 50 °C (Figure S4C). The enzyme maintained 90% of its activity at 55 °C, but the activity declined sharply to 30% at 60 °C. Additionally, OPAA114644 exhibited moderate thermostability (Figure S4D). The enzyme showed no loss of activity after incubation at 50 °C for 1 h and maintained more than 75% of its initial activity after incubation at 55 °C for 1 h. However, the enzyme lost almost all its activity after incubation at 60 °C for 15 min. The profiles of pH and temperature of OPAA114644 were similar to OPAAs from *Alteromonas* species, whose optimal pH and temperature ranges were 7.5–8.5 and 40–55 °C, respectively [34].

In practical application, enzymes are inevitably exposed to salt solution; thus, enzymes with high salt tolerance are advantageous for further use. The salt tolerance of OPAA114644 was analyzed (Figure S4E). The activity of OPAA114644 tripled in the presence of 10% NaCl and continued to increase nearly fourfold in 20% NaCl. In the presence of 30% NaCl, 64% of the initial activity was retained. The mechanism by which high-concentration salt could enhance OPAA114644 activity is unclear. Enzymatic properties are closely related to the environment in which the enzyme is located. The high salt tolerance of OPAA114644 may be attributed to its origin in the ocean, which contains a high amount of salt. Few studies have reported the effect of salt on OPAA activity, which includes OPAA from halophilic bacteria *Alteromonas* sp. JD6.5. *Alteromonas* sp. JD6.5 was isolated from a warm salt spring with a salt concentration of approximately 24% [35]. The high salt tolerance of OPAA114644 not only makes it advantageous for industrial application, but also for the biotreatment of OP pesticide pollution in high-salt environments.

Because of the insolubility of OP compounds in water, it is important for OPAAs to tolerate organic solvents in practical applications. The effects of organic solvents on OPAA114644 were thus assessed (Figure S4F). It was found that 10% (*v*/*v*) methanol had a moderate inhibitory effect on OPAA114644 activity, while ethanol, acetone, acetonitrile, and DMSO significantly inhibited the activity. Moreover, a remarkable inhibitory effect was observed in the presence of 1 mM SDS and EDTA (Figure S4G). The strong inhibitory effect of EDTA on enzymatic activity, on the other hand, verified the importance of metal ions in OPAA114644 activity. OPAA114644 activity was slightly inhibited by 0.1% β-mercaptoethanol and Triton X-100, while DTT and Tween 20 had a limited effect on the activity (Figure S4G).

### 3.6. Metal Ions Analysis

**Figure 5:**
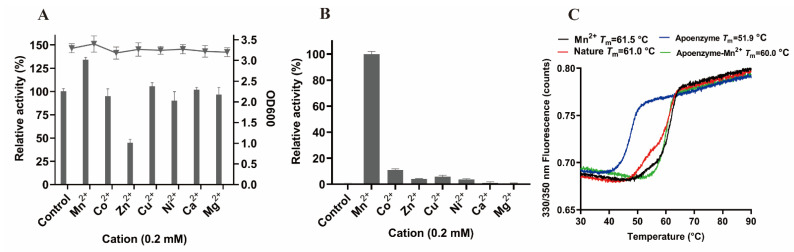
Metal ions analysis. (**A**) Effects of different metal ions in medium on enzyme activity (bar graph) and OD600 (line chart). Control without any metal ion in medium was set as 100% of the activity. (**B**) Effects of different metal ions on apoenzyme activity. The control was apoenzyme, and the activity with Mn^2+^ was set as 100%. (**C**) Effect of Mn^2+^ on the *T*_m_ of OPAA114644. The samples included pure enzyme with (black) or without (red) 0.2 mM Mn^2+^ in medium, apoenzyme (blue), and apoenzyme reconstructed with 0.2 mM Mn^2+^ (green).

As metalloenzymes, the activities of OP-degrading enzymes depend on metal ions bound to the active center [12]. Studies have reported that the levels of PTE activities are dependent on the presence of divalent cations in the growth medium, which could promote protein folding [25,26,36]. Divalent cations were introduced into the growth medium to explore the effects of metal ions on OPAA114644 activity (Figure 5A). Different metal ions were introduced into the medium and had no effect on the cell density of *E. coli*. However, only the introduction of Mn^2+^ increased the enzyme activity by 1.35 fold compared with the unsupplemented medium, and the addition of other metal ions did not affect the activity, expect for Zn^2+^, which inhibited the activity by 45% in relation to that of the unsupplemented medium. LB medium provided a specific amount of metal ions for the OPAA114644. The metal ion substitution experiments on the apoenzyme revealed that the loss of metal ions could completely inactivate the enzyme, and only Mn^2+^ could reactivate the apoenzyme (Figure 5B), suggesting that Mn^2+^ was essential and irreplaceable for OPAA114644 activity.

The circular dichroism spectrum analysis (Figure 3C) indicated that the profiles of apoenzyme and apoenzyme reconstructed with 0.2 mM Mn^2+^ were the same as that of the native OPAA114644, indicating that Mn^2+^ had no effect on the secondary structure of OPAA114644.

The effect of Mn^2+^ on OPAA114644 *T*_m_ was evaluated through differential scanning fluorimetry (Figure 5C). The addition of Mn^2+^ in the medium increased OPAA114644 *T*_m_ from 61.0 °C to 61.5 °C. The *T*_m_ of the apoenzyme dropped to 51.9 °C, and the re-addition of Mn^2+^ restored the *T*_m_ of the apoenzyme to 60.0 °C. Notably, the addition of Mn^2+^ to the apoenzyme failed to recover the *T*_m_ to 61.0 °C. This might have been caused by Mn^2+^ deficiency. It is also possible that EDTA chelation led to structural disorder, which affected the binding efficiency between Mn^2+^ and the apoenzyme, and subtle structural changes could not be detected using a circular dichroism spectrometer. A high *T*_m_ value indicates a stable structure [37]. Therefore, Mn^2+^ ions are not only necessary for the catalytic activity but also involved in OPAA114644 structure stabilization. The same phenomenon was observed in MPH from *Azohydromonas australica* [24] and prolidase in human [38].

### 3.7. Decontamination of Paraoxon on Glasses, Cotton Tissues, and Apples by OPAA114644

The decontamination experiments of paraoxon on different surfaces such as glasses, cotton tissues, and apples by OPAA114644 were performed to assess the application potential of the enzyme in the pesticide decontamination field.

**Figure 6:**
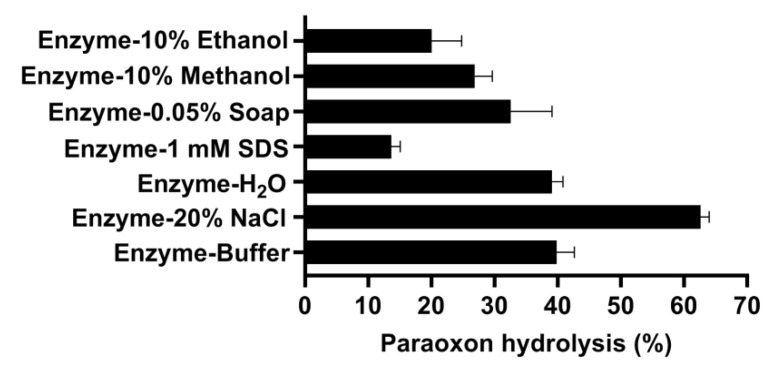
Decontamination experiments of glass surfaces in different enzyme solutions. Glasses were contaminated by 10 nmol paraoxon and decontaminated by 10 µL OPAA114644 at a concentration of 2 mg/mL in different solutions.

First, the glass surfaces were contaminated with 10 nmol paraoxon and decontaminated with different enzyme solutions, including only tap water, only buffer, or buffer with 20% NaCl, 1 mM SDS, 0.05% soap, and 10% methanol or 10% ethanol, to mimic the conditions in daily washing or in a pressurized and denatured industrial environment (Figure 6). After incubation for only 10 min at room temperature, 62% of paraoxon could be degraded from the glass surface in the enzyme solution with 20% NaCl. Interestingly, in the presence of only buffer and only tap water, about 40% of paraoxon was removed. In the enzyme solutions containing 0.05% soap, 10% methanol and 10% ethanol, 32%, 26%, and 20% of paraoxon was degraded, respectively. In the presence of 1 mM SDS, only 14% of paraoxon was degraded.

**Figure 7:**
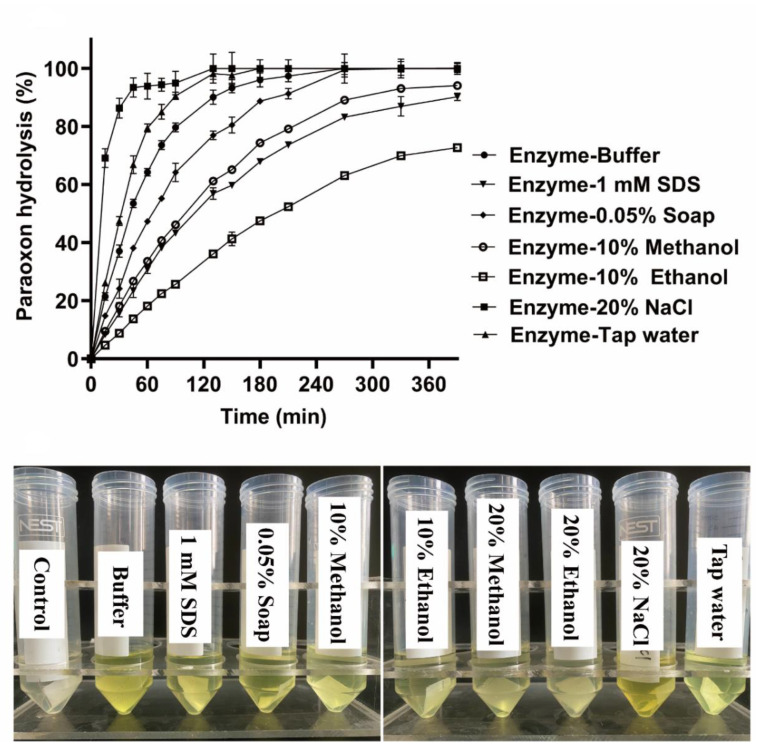
Decontamination experiments of cotton tissues in different enzyme solutions. Cotton tissues were contaminated by 0.5 µmol paraoxon and decontaminated by 100 µg/mL OPAA114644 in different solutions at room temperature.

Similar decontamination experiments were performed using cotton tissues (2 × 2 cm^2^) contaminated with 0.5 µmol paraoxon in the different enzyme solutions described above. As shown in Figure 7, 93% of paraoxon was removed by 100 µg/mL OPAA114644 in the presence of 20% NaCl after only 45 min, and paraoxon was completely degraded after 130 min. Interestingly, OPAA114644 exhibited a higher degradation rate towards paraoxon in tap water (100%, 180 min) than in buffer solution (100%, 270 min). Paraoxon could be completely removed in the presence of 0.05% soap after 330 min. About 90% of paraoxon was degraded in the presence of 10% methanol and 1 mM SDS after 270 and 390 min, respectively. OPAA114644 could remove 73% of paraoxon in the presence of 10% ethanol after 390 min.

**Figure 8:**
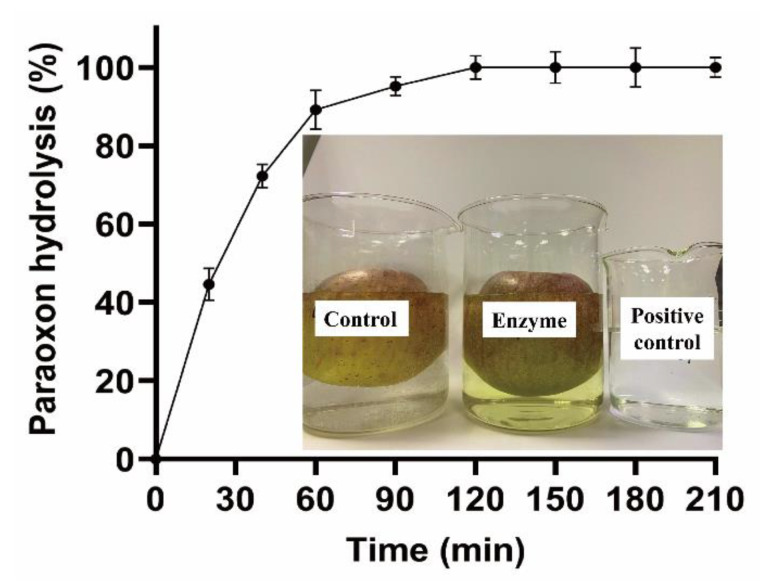
Decontamination experiments of apples in tap water. Apples were contaminated by 1.5 µmol paraoxon and decontaminated by 50 µg/mL OPAA114644 in tap water at room temperature.

Fruit decontamination experiments were performed by washing apples contaminated with 1.5 µmol paraoxon with tap water containing the enzyme (Figure 8). About 90% of paraoxon was degraded after 60 min, and paraoxon could be completely removed from washing solution and apples by 100 µg/mL OPAA114644 after 120 min. About 60% of paraoxon could be washed from apples by tap water without enzyme and remained in the washing solution, so paraoxon can still diffuse into the environment with toxic effects. The decontamination of OPAA114644 solves this problem.

The results of these decontamination experiments demonstrated that OPAA114644 could effectively degrade paraoxon on the surfaces of glasses, cotton tissues, and apples in different solutions. In the presence of 20% NaCl, OPAA114644 showed the highest degradation efficiency towards paraoxon on glasses and cotton tissues. Notably, the degradation rate of OPAA114644 towards paraoxon in only tap water was not lower than that in only buffer, and it was higher in only tap water than in only buffer in the decontamination experiments on cotton tissues. Similarly, OPAA114644 also showed effective decontamination in the washing experiments of paraoxon on apples in tap water, proving that OPAA114644 has a good prospect in the decontamination of foods, clothes, and floors in daily life. In daily life and food processing plants, contact with detergents and organic solvents is inevitable, so it is necessary to test the decontamination of the enzyme in the presence of detergents and organic solvents. Although the degradation rates of OPAA114644 in the presence of 1 mM SDS, 0.05% soap, 10% methanol, and 10% ethanol were lower than that in the presence of tap water and only buffer, it could still effectively degrade the majority of residual paraoxon on the surfaces of cotton tissues over time, which makes it possible that the enzyme can be used together with detergents during daily washing.

There are some reports on the decontamination of organophosphorus-degrading enzymes towards pesticides on the surfaces of fruits, cotton tissues, and so on. One of them is about the mutated phosphotriesterase (*Po*OPH_M9_) expressed by *Pichia pastoris*, which could completely degrade 12 µmol malathion on apple and cucumber surfaces in 2 L tap water at the concentration of 50 µg/mL after 30 min washing [39]. Giudice et al. [27] obtained a mutant of SsoPox from *Sulfolobus solfataricus* through semi-rational evolution in vitro, which could quickly and effectively degrade the paraoxon on the surfaces of glasses, cotton tissues, and apples. Furthermore, they proved that the degradation rates depended on the amounts of pesticides and enzymes used. OPAAs exhibit a higher activity towards G-type nerve agents than OP pesticides, and there are few reports on the decontamination of OPAA towards pesticides on foods, clothes surfaces, and so on. The effective decontamination of OPAA114644 towards paraoxon on the surfaces of glasses, cotton tissues, and apples provides a real application for the decontamination of foods and clothes and for the detoxification/remediation of sites, such as floors, using OPAA.

## 4. Conclusions

In this work, we discovered a novel OPAA from deep-sea sediment. OPAA114644 was successfully expressed and purified. The pure enzyme exhibited excellent alkali stability over a wide pH range of 5.0–9.0, high salt tolerance, and high degradation efficiency for soman, dichlorvos, paraoxon, and coumaphos. Notably, OPAA114644 dissolved in different solutions, such as 20% NaCl, 1 mM SDS, 0.05% soap, 10% methanol, and tap water, could efficiently decontaminate the residual paraoxon on the surfaces of glasses, cotton tissues, and apples, indicating its excellent potential for the biodegradation and decontamination of OPCs pollution. With the growing concern about food safety and environmental pollution caused by pesticides, economical, efficient, and environmentally friendly methods for pesticide degradation are urgently needed, and the effective decontamination of paraoxon on the surfaces of glasses, cotton tissues, and apples using OPAA114644 represents a real application of enzymes in the decontamination/detoxification of foods and clothes and in the bioremediation of sites such as floors.

The results also illustrate the potential benefits of enzymes from an extreme deep-sea environment in the biotreatment of OP pesticide pollution; deep-sea sediment might be an abundant resource for various microorganisms and enzymes. In addition, the potential of OPAA114644 deserves further exploration, such as using protein engineering technology to improve the thermostability and resistance to organic solvents. It also can be used to develop a biosensor to detect OP pesticides and G-type nerve agents, which needs to be studied further.

## Figures and Tables

**Table 1:** Kinetic parameters of OPAA114644 for Met-Pro and paraoxon.

Substrate	*K*_m_ (mM)	*V_max_* (µmol/min/mg)	*k*_cat_ (s^−1^)	*k*_cat_/*K*_m_ (mM^−1^ s^−1^)
Met–Pro	2.72 ± 0.66	11,295 ± 1102	5445	2002
Paraoxon	2.06 ± 0.19	2.22 ± 0.07	0.75	0.36

## Data Availability

Not applicable.

## Supplementary Materials

The following supporting information can be downloaded at: https://www.mdpi.com/article/10.3390/microorganisms10061112/s1, Table S1: Characteristics of the organophosphorus-degrading enzymes. Figure S1: Chemical structures and mass spectrums of OP pesticides and nerve agent. Figure S2: Activity of OPAA114644 against Xaa-Pro dipeptides. The final concentration of Xaa-Pro dipeptides was 4 mM, and the relative activity for Met-Pro was set as 100%, corresponding to specific activity of 3095 U/mg. The values are an average of three independent experiments, and error bars represent standard deviation. Figure S3: Kinetic analysis of OPAA114644 for paraoxon and Met-Pro (fitted to the Michaelis–Menten kinetics). (A) Michaelis–Menten kinetics for paraoxon. (B) *k*_cat_ for paraoxon. (C) Michaelis–Menten kinetics for Met-Pro. (D) *k*_cat_ for Met-Pro. Figure S4: Biochemical characterization of OPAA114644. (A) Effects of pH on enzyme activity. (B) Effects of pH on enzyme stability. (C) Effects of temperature on enzyme activity. (D) Thermostability. (E) Effects of NaCl on enzyme activity. (F) Effects of organic solvents on enzyme activity. (G) Effects of other chemical agents on enzyme activity. Control was set as 100% of activity without NaCl, organic solvents, or other chemical agents in the mixture reaction. The values are an average of three independent experiments.
