# Supplementary material for: A Novel Organophosphorus Acid Anhydrolase from Deep Sea Sediment with High Degradation Efficiency for Organophosphorus Pesticides and Nerve Agent
Source: Microorganisms. 2022 May 27;10(6):1112. doi: 10.3390/microorganisms10061112 (PMC9231299; doi:10.3390/microorganisms10061112)
Supplement: Supplementary file 1 [file microorganisms-10-01112-s001.zip › microorganisms-1714764-supplementary.pdf]

microorganisms

MDPI

Supplementary materials

A Novel Organophosphorus Acid Anhydrolase from Deep Sea Sediment with High Degradation Efficiency for Organophosphorus Pesticides and Nerve Agent

Nucleotide sequence

ATGGAAGCTAACAATAACTATTCTGCACATATTGCAGAACTTCAAC-
GCAGAACTAAAAAAGCCTTAGCACGAGAAAATTTAGATGCTTT-
GGTGATCCACTCAGGCAAAGAAATTAAAGTTTTTTTAGACGAC-
TATAGTTACCCTTTCAAAGTTAACCCTCATTTTAAAGCTTGGTTGCCAATAAC-
CGATGTTGCTAACTGTTGGCTTATTGTAAATGGCGAAGATAAACCTACAC-
TTATTTATTACCAACCTATTGATTTTTGGCATAAAGTAATTGAATTAACAGA-
CAGTTACTGGCAAGAGTTTTTTGATATTAAAATATTAAAAGTT-
GCCAGCGAAGTGGATAAGCTTTTACCTTATGATAAAAAAGGTTATGCC-
TACATAGGTGAGCATATTGAAGTTGCTAAAGCCTTAGGATTTGAAGCAA-
TAAATCCAGAGCCTTTAATGAATTA-
TATGCATTATCATCGAGCTTATAAAACAGCTTATGAATTT-
GATTGTATGCGAAAATCTAACGCGTTAGCGGTAAAGGCTCATCAAGCGG-
CACAACAAGCTTTTTTAGCGGGTGAAAGTGAATTTGCTATTCAACAA-
GCTTATTTAAAGGCTATCGCTTATTCAGAAAATGATACACCTTATGGCAA-
TATTGTTGCGCTTAATGAAAATGCAGCAATATTACATTACACCGCTTTAGTTCG-
GAACAAACCTCAAGAACCACGGTCATTTTTAATT-
GATGCGGGCGCTAATTTTAATGGTTATGCTGCTGACATTACACGCAC-
TTATGCTTTTAAACAAAATAAATTTGCAGAATTAATTGCTCGTATGGATCAG-
TTAATGCTTAAAGCTGTTGATGGCTTAAAGCCAAATAAAAGCTATGTAGAT-
TTACATATTGAAACTTATGCGGAAATAGGCAAGGTTTTAAAAGAATTTGAT-
TTTATTAATGTTGAAGCAGACACGGCAGTGGAAACAGGGAT-
TATTTCAACATTTTTTCCGCATGGTTTAGGG-
CATCATTTAGGTTTACAAACCCATGATGTTGGTGGCTTTATGGCCGATGAAC-
GAGGCACACATATTAATGCACCTAAAAATCATCCTTTTTTAA-
GAACTTCTCGAGTTATTGAAGCTAATCAAGTATTTACTATTGAACCGGGCTT-
GTATTTTATCGATTCACTACTTAAAGAGCTGAAACAGTCAAAA-
TATGCACAAATGGTAAATTGG-
CAAAAAGTAGATGAAATGCGTCCTTATGGGGGCATTCGTATAGAAGATAA-
TATTATTGTTCATAAAAATCATAATGAAAATATGACACGCGAAGCAGGAT-
TAAATTAA

Amino acid sequence

MEANNNYSAHIAELQRRTKKALARENLDALVIHSGKEIKVFLDDY-
SYPFKVNPHFKAWLPITDVANCWLIVNGEDKPTLIYYQPIDFWHKVIELT-
DSYWQEFFDIKILKVASEVDKLLPYDKKGYAYIGEHIEVAKALGFEAINPEPLM-
NYMHYHRAYKTAYEFDCMRKSNALAVKAHQAAQQAFLAGESEFAIQQAYLKA-
IAYSENDTPYGNIVALNENAAILHYTALVRNKPQEPRSFLIDAGANFNGYAADIT-
RTYAFKQNKFAELIARMDQLMLKAVDGLKPNKSYVDLHIETYAEIGKVLKEFDFIN-
VEADTAVETGIISTFFPHGLGHHLGLQTHDVGGFMADERGTHINAP-
KNHPFLRTSRVIEANQVFTIEPGLYFIDSLLKELKQSKYAQMVNWQKVDEMRPYGGI-
RIEDNIIVHKNHNENMTREA

Table S1. Characteristics of the organophosphorus-degrading enzymes[a].

Enzyme	OPH	OpdA	MPH	OPAA	OPN 1	DFPase
**Organism source**	*Brevundimonas diminuta; Flavobacterium* sp.	*Agrobacterium radiobacter*	*Pseudomonas* sp. WBC3; *Plesiomonas* sp. strain M6	*Alteromonas* sp. strain JD6.5	Mammalian serum	*Lolilgo vulgaris* squid
**Structure**	$(\alpha/\beta)_8$-barrel fold	$(\alpha/\beta)_8$-barrel fold	β-Lactamase fold	Pita bread fold	β-Propeller fold	β-Propeller fold
**Molecular Weight**	36 kDa	35 kDa	34 kDa	58 kDa	43 kDa	35 kDa
**Metals**	Zn^{2+}, Co^{2+}, Ni^{2+}, Cd^{2+}, Mn^{2+}	Fe^{2+}, Zn^{2+}, Co^{2+}	Co^{2+}, Ni^{2+}, Mn^{2+}, Zn^{2+}, Fe^{2+}, Cd^{2+}	Mn^{2+}, Co^{2+},	Ca^{2+}	Mg^{2+}, Ba^{2+}, Ca^{2+}
**Substrate**	Paraoxon, Parathion, Malathion, Chlorpyrifos, Demeton-S, Diazinon, Coumaphos, DFP, VX, VR, GA, GB, GD, GF	Paraoxon, Parathion, Diazinon, Coumaphos, GA; GB; GD	Methyl-parathion, Parathion, Paraoxon	Paraoxon, DFP, GD, GB, GA	Lactones, Paraoxon, GD	DFP, GD, GB, GA
**Optimum pH**	9.0	9.0	9.0	8.5	8.0	8.0
**Optimum temperature**	35 °C	40 °C	37 °C	45 °C	45 °C	30°C
**PDB ID**	1DPM; 1EZ2; 1HZY	2R1N	1P9E	3L7G	1V04	2GVV

[a] represents the cited literature [6].

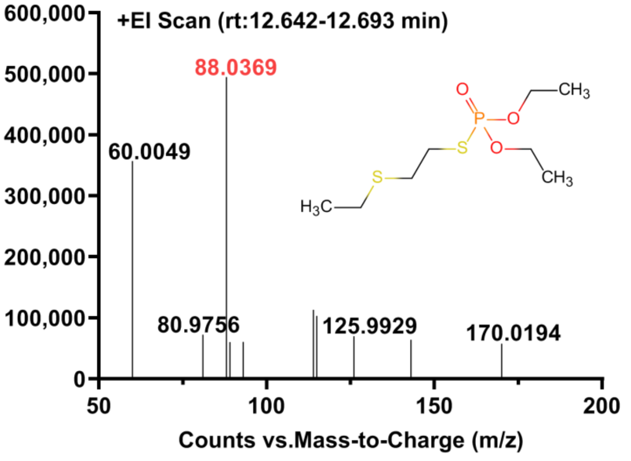

(**a**) Demeton-S

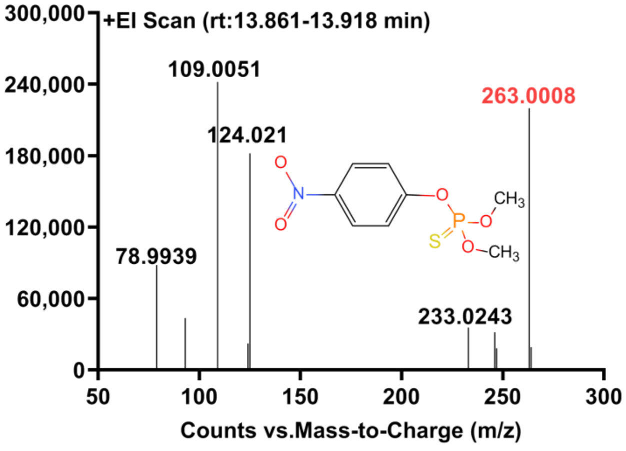

(**b**) Parathion-methyl

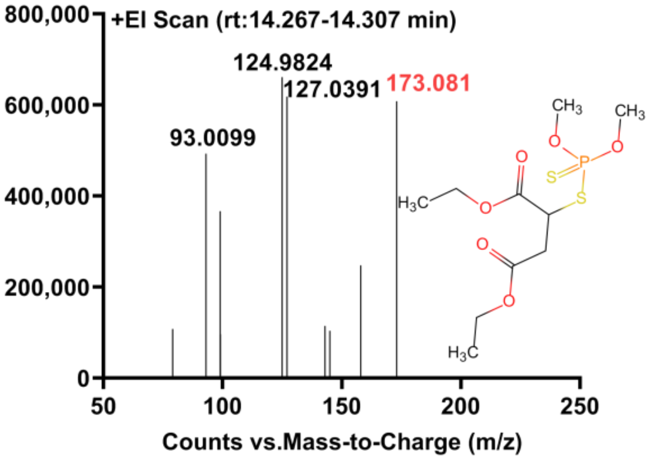

(**c**) Malathion

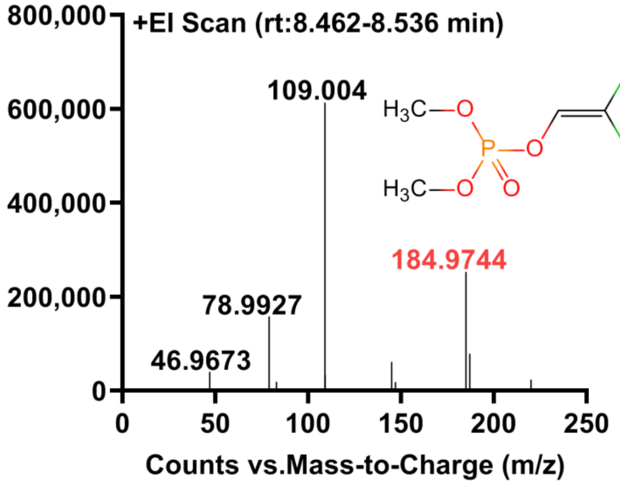

(**d**) Dichlorvos

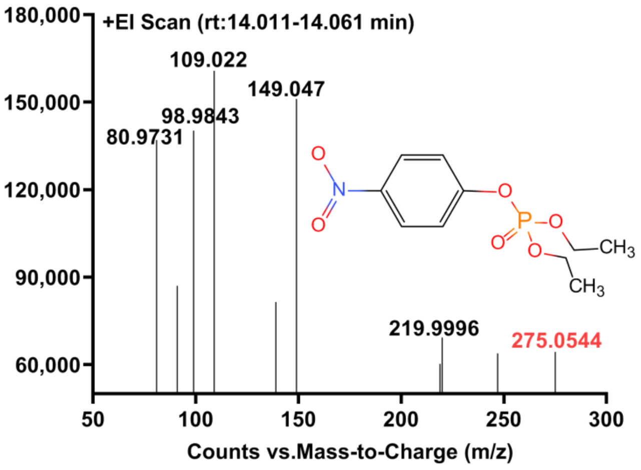

(**e**) Paraoxon

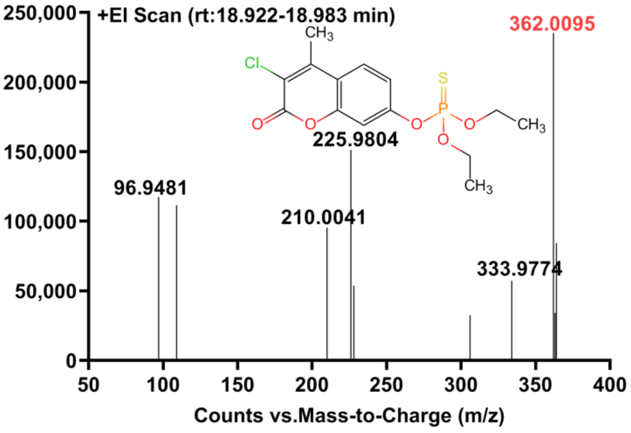

(**f**) Coumaphos

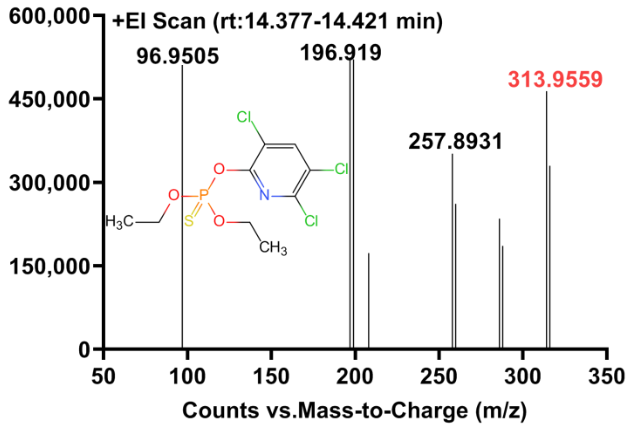

(**g**) Chlorpyrifos

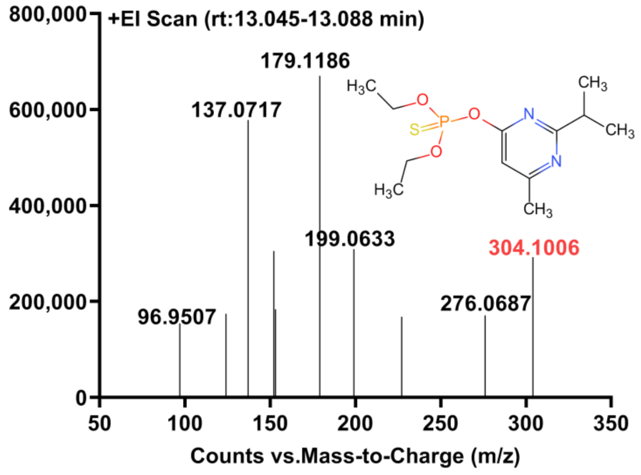

(**h**) Diazinon

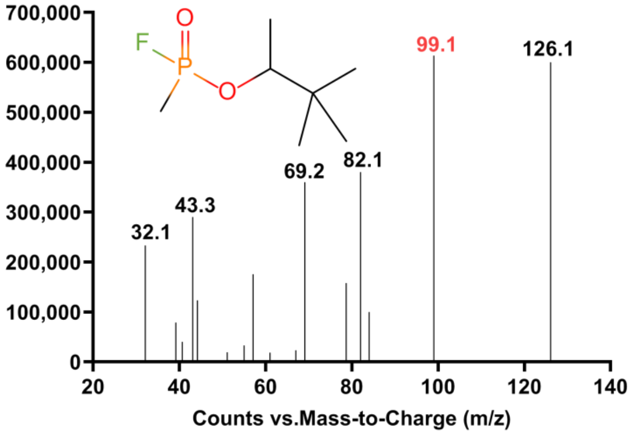

(i) Soman

Figure S1. Chemical structures and mass spectrums of OP pesticides and nerve agent.

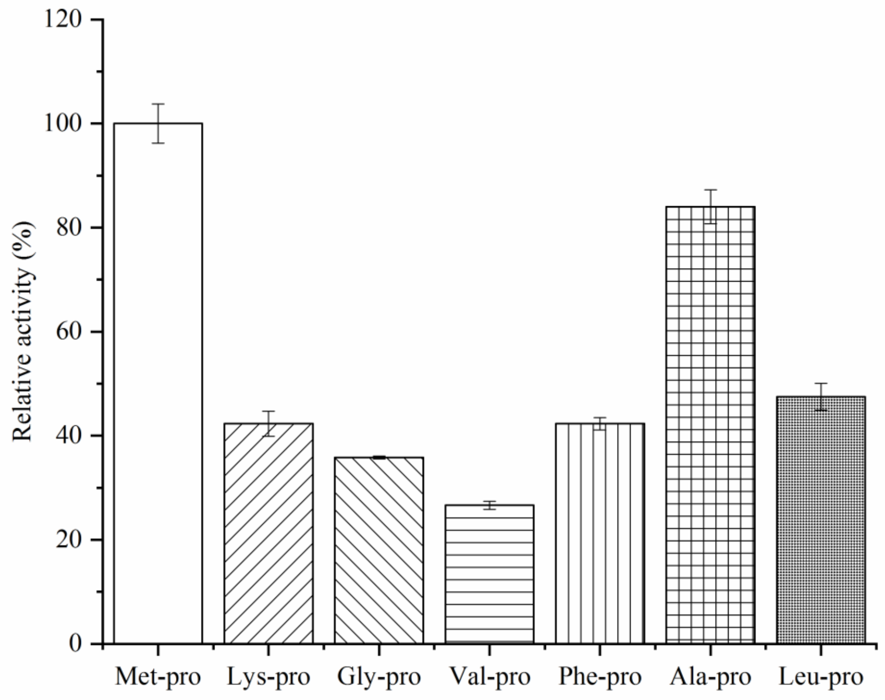

Figure S2. Activity of OPAA114644 against Xaa-Pro dipeptides. The final concentration of Xaa-Pro dipeptides was 4 mM, and the relative activity for Met-Pro was set as 100%, corresponding to specific activity of 3095 U/mg. The values were an average of three independent experiments and error bars represented standard deviation.

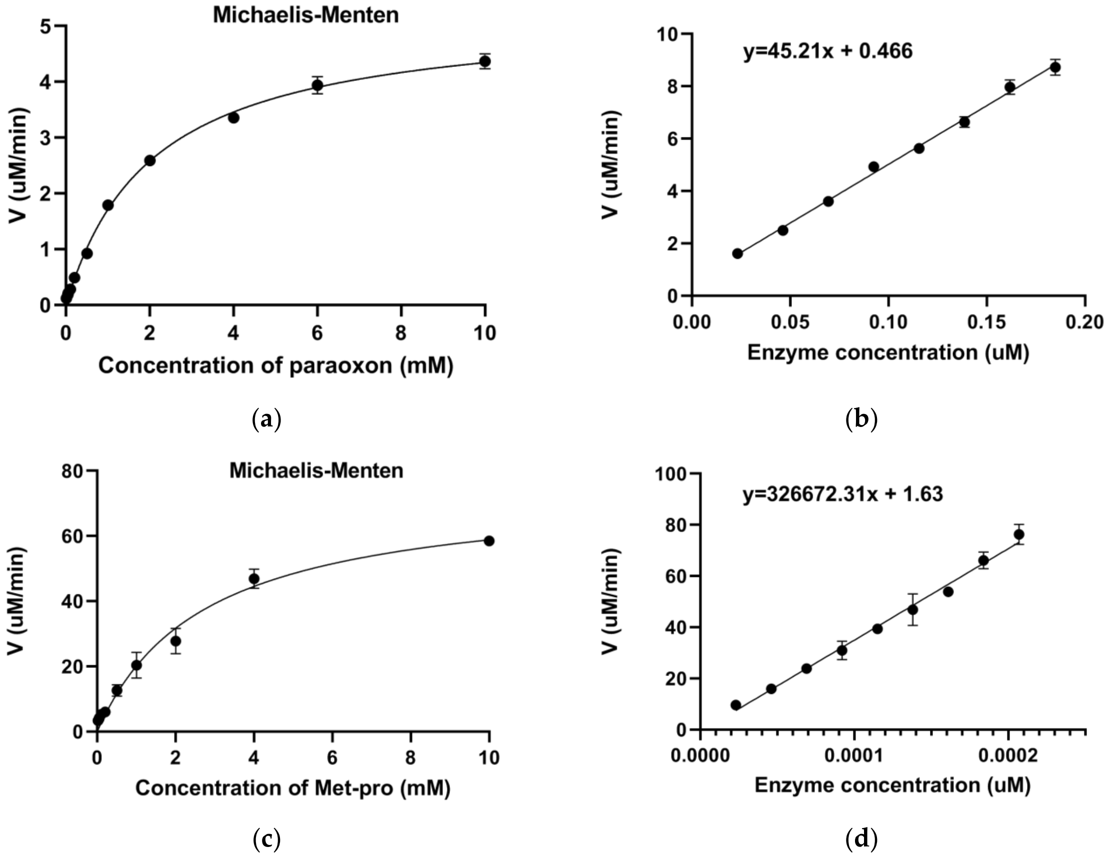

Figure S3. Kinetic analysis of OPAA114644 for paraoxon and Met-Pro. (fitted to the Michaelis-Menten kinetics). (**A**) Michaelis-Menten kinetics for paraoxon. (**B**) k_{cat} for paraoxon. (**C**) Michaelis-Menten kinetics for Met-Pro. (**D**) k_{cat} for Met-Pro.

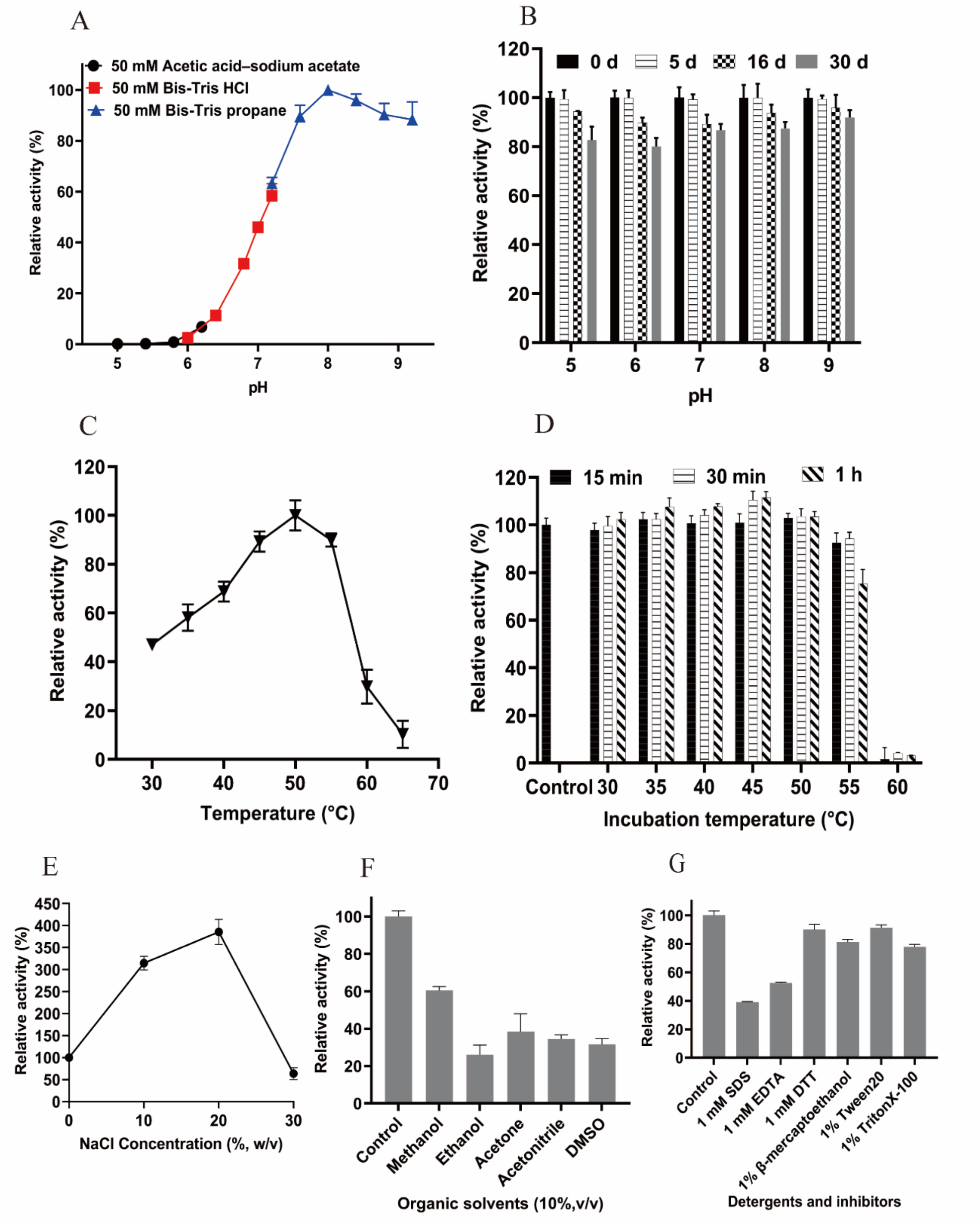

Figure S4. Biochemical characterization of OPAA114644. (**A**) Effects of pH on enzyme activity. (**B**) Effects of pH on enzyme stability. (**C**) Effects of temperature on enzyme activity. (**D**) Thermostability. (**E**) Effects of NaCl on enzyme activity. (**F**) Effects of organic solvents on enzyme activity. (**G**) Effects of other chemical agents on enzyme activity. Control was set as 100% of activity without NaCl, organic solvents and other chemical agents in the mixture reaction. The values were an average of three independent experiments.
